# Chronic effects of high-intensity functional training on motor function: a systematic review with multilevel meta-analysis

**DOI:** 10.1038/s41598-020-78615-5

**Published:** 2020-12-10

**Authors:** Jan Wilke, Lisa Mohr

**Affiliations:** Department of Sports Medicine, Goethe University, Frankfurt, Frankfurt/Main, Germany

**Keywords:** Medical research, Preventive medicine

## Abstract

High-intensity functional training (HIFT) has become a popular method in the sports and fitness sector. In contrast to unimodal approaches such as strength or endurance training, it has been hypothesized to induce concurrent adaptations in multiple markers of motor function. However, to date, the effectiveness of HIFT in this regard has not been studied. The present systematic review quantified the chronic effects of HIFT on motor function in healthy individuals. A multilevel meta-analysis with a robust random effects meta-regession model was used to pool the standardized mean differences (SMD) between (a) HIFT and (b) no-exercise (NEX) as well as conventional endurance, resistance and balance training for outcomes of muscle strength, endurance capacity and balance. The influence of possible effect modifiers such as program duration, session duration, age or sex was examined in a moderator analysis. Seventeen papers with moderate to high methodological quality (PEDro scale) were identified. Compared to NEX, HIFT had small to moderate positive effects on endurance capacity (SMD: 0.42, 95% CI 0.07–0.78, p = 0.03) and strength (0.60, 95% CI 0.02–1.18, p = 0.04) but no effect on balance (SMD: − 0.10, 95% CI − 1.13 to 0.92, p = 0.42). Regarding endurance, HIFT showed similar effectiveness as moderate-intensity endurance training (SMD: − 0.11, 95% CI − 1.17 to 0.95, p = 0.75) and high-intensity interval endurance training (SMD: − 0.15, 95% CI − 1.4 to 1.1, p = 0.66). No comparisons of HIFT vs. classical resistance or balance training were found. Moderator analyses revealed no influence of most effect modifiers. However, regarding endurance, females seemed to respond more strongly to HIFT in the comparison to NEX (p < .05). HIFT appears to represent an appropriate method to induce chronic improvements in motor function. While being superior to NEX and non-inferior to endurance training, current evidence does not allow a comparison against resistance and balance training. The impact of possible effect moderators should be further elucidated in future research.

## Introduction

High-intensity functional training (HIFT) has become a popular trend in the sports and fitness sector^1^. It is commonly characterized as the strenuous performance of exercises mimicking movements of daily life (e.g. squats, lunges or push-ups) interspersed with short breaks^2^. Contrary to high-intensity training, which is rather unidimensional and typically focused on one motor ability (e.g. running or cycling to improve endurance), HIFT aims to integrate cardiovascular, neuromotor and muscular efforts which is achieved by a variety of strategies such as the selection of whole-body exercises maximizing oxygen consumption, fast movement execution and the optional use of scalable weights (e.g. dumbbells, medicine balls, resistance bands).

HIFT may have relevant advantages for different populations. Many inactive individuals report a lack of time to represent a significant barrier to engaging in physical activity^3,4^. HIFT workouts tend to have short durations of mostly below 30 min and may thus be more appealing than conventional programs with longer durations^2^. In addition, stronger increases in intrinsic motivation predicting long-term activity adherence have been observed following HIFT when compared to continuous, moderate-intensity exercise^5,6^. While these data render HIFT an interesting option for sedentary individuals, it may also be of interest for athletes. Analyses of team sports show that many markers of motor function (e.g. strength, running endurance, postural control) are not or only weakly predictive of performance when considered in isolation^7,8^. This may be due to the fact that most sports do require a fine-orchestrated combination of motor skills. Owing to its multimodal nature, HIFT’s ecological validity for sporting performance may be higher than that of traditional approaches.

In order to gauge the potential of HIFT in exercise counseling for inactive individuals and program design for athletes, its effectiveness needs to be compared to classical exercise regimes. Despite the existence of related trials with an appropriate study design (RCT and crossover trials), there is no quantitative data synthesis pooling their findings using meta-analytic techniques. Furthermore, it is unknown, which variables moderate the potential effects of HIFT on parameters of motor function. The present systematic review with meta-analysis, therefore aimed to investigate the effects of HIFT on motor performance as compared to classical training methods.

## Methods

A systematic review with multilevel meta-analysis and a random effects meta-regression model was performed. It adhered to the PRISMA (Preferred Reporting Items for Systematic Reviews and Meta-Analyses) guidelines^9^ and followed the recommendations for ethical publishing of systematic reviews proposed by Wager and Wiffen^10^. Prior to its initiation, the study was registered in the PROSPERO database (CRD42020170412).

### Search strategy

Between February and March 2020, two independent investigators (JW, LM) performed a systematic literature search. Articles matching the research question were identified using MEDLINE (PubMed), CochraneCentral, Web of Science and Google Scholar*.* The terms for all databases were similar but modified according to the requirements of the respective search masks. As an example, the term used for PubMed was: ‘high-intensity AND (functional OR body weight) AND (exercise OR training OR workout OR circuit OR conditioning) NOT acute’. For Google Scholar, an approach described in previous systematic reviews of our work group^11–13^ was used. Briefly, the first 100 hits displaying the most relevant findings in regards to the entered term were screened for relevant articles in order to complement the results of the other database searches. In addition, the reference lists of all included studies were checked in order to identify further potentially eligible papers^14^.

### Inclusion criteria

Randomized controlled trials (crossover or parallel group design) with accessible full text were considered for inclusion. Further criteria were (1) enrolment of healthy individuals, (2) performance of high-intensity functional exercise, (3) testing of chronic effects (minimum of 4 weeks training) on markers of strength, endurance or balance (4) control against inactivity, strength, endurance or balance exercise as well as (5) publication in English language and in a peer-reviewed journal. Interventions were classified as HIFT if they were performed at high relative training intensities, aimed to improve multiple motor functions (e.g. strength, endurance, balance) and included multiple different functional whole-body movements (e.g. jumps, squats, burpees, push-up, running in place). All studies investigating acute effects or other training methods (including combined treatments), lacking the control group types listed in (4) or including persons with diseases were excluded.

### Data extraction

Using a standardised assessment sheet, two investigators (JW, LM) independently extracted the following data from included papers: study design, sample size, participant characteristics, interventions including their characteristics (see below), measured outcomes and results (pre-post changes plus standard deviations of each intervention arm). Outcomes of the meta-analysis were strength, endurance and balance. If a study reported more than one strength (e.g., shoulder and leg), endurance (e.g. Vo^2^max and Bruce test) or balance measure, all respective effect sizes (ES) were extracted.

### Data synthesis and statistics

Data from both crossover and parallel-group trials were included. For each intervention arm of parallel-group studies, the mean pre- to post-test changes plus standard deviations (SD) were retrieved. If reporting was incomplete (i.e. missing SDs of the changes from baseline), the required information was requested from the corresponding authors of the trials. If no values could be obtained, missing data were (1) determined from figures or p values/t values/standard errors or (2) imputed according to the recommendations of the Cochrane handbook, using the formula SD_change_ = √(SD^2^_baseline_ + SD^2^_postintervention_)–(2 × Corr × SD _baseline_ × SD _postintervention_), where Corr = 0.7. The value chosen for Corr represents a conservative estimate of the correlation between the baseline and post-treatment SDs^15^. For crossover trials, the SD of the difference between the two relevant condition’s pre-post changes, the correlation of the respective pre-post changes as well as the standard error were computed. If the correlation coefficient for the conditions’s pre-post changes could not be extracted from publications or calculated from raw data, a conservative value of 0.5 was assumed, which also fits with the known correlations of the other included studies. When combining the results from parallel group and crossover studies, we used appropriate formulae for standardized mean ES and standard errors^16^.

The following potential moderators of the treatment effect were dichotomized (for details refer to tables): program duration (weeks), session duration (mins), total program volume (mins), rest interval duration (seconds), age (years) and sex (female and male). The choice of the tested moderators was based on three criteria^17^. Firstly, they had to be clearly reported in at least five studies. Secondly, variation had to be present between the levels of a moderator. For instance, if all studies would have stated the sex of the participants, a moderator analysis would have been impossible if only males were included in these studies. Finally, there had to be a plausible theory as to how a moderator would influence the treatment outcome. For instance, it may be assumed that age, with its two moderator levels old and young would lead to a varying treatment effectiveness.

A multilevel meta-analysis with a robust random effects meta-regression model^18^ was used to pool the standardized mean differences (SMD) and 95% confidence intervals (CI) between HIFT and no-exercise control (NEX), HIFT and moderate continuous aerobic training (MCT), HIFT and high-intensity interval training (HIIT), HIFT and resistance training (RES) as well as HIFT and balance training (BAL). Dependency of ES was taken into account by nesting the term ‘study’ as a random factor in the model. Potential moderators were identified with separate models: (1) estimating the significance of each level by means of the 95% CI and (2) testing for differences between the respective levels^13,19^. The between-study variance component was determined by means of Tau^2^, using the method-of-moments estimate; for within-study variance (more than one dependent effect size), omega^2^ (ω^2^) was calculated^18^. Pooled effect sizes were interpreted as small (SMD = 0.2), modreate (SMD = 0.5) or large (SMD = 0.8)^20^. P values < 0.05 were considered significant. The software employed was R (R Foundation for Statistical Computing, Vienna, Austria), packages meta (G Schwarzer) and robumeta (version 2.0^21^).

### Risk of bias and study quality

Publication bias was checked by means of visual inspection of funnel plots (ES against standard errors) and optional sensitivity analyses excluding outliers if at least 10 ES were available^22^. The methodological quality of the included studies was assessed by means of the PEDro scale, which has been shown to represent a high reliability and validity for this purpose^23–25^. The sum score of the instrument is calculated from 10 items capturing potential sources of bias. Two independent investigators (JW, LM) performed the quality scoring.

## Results

### Search results

**Figure 1:**
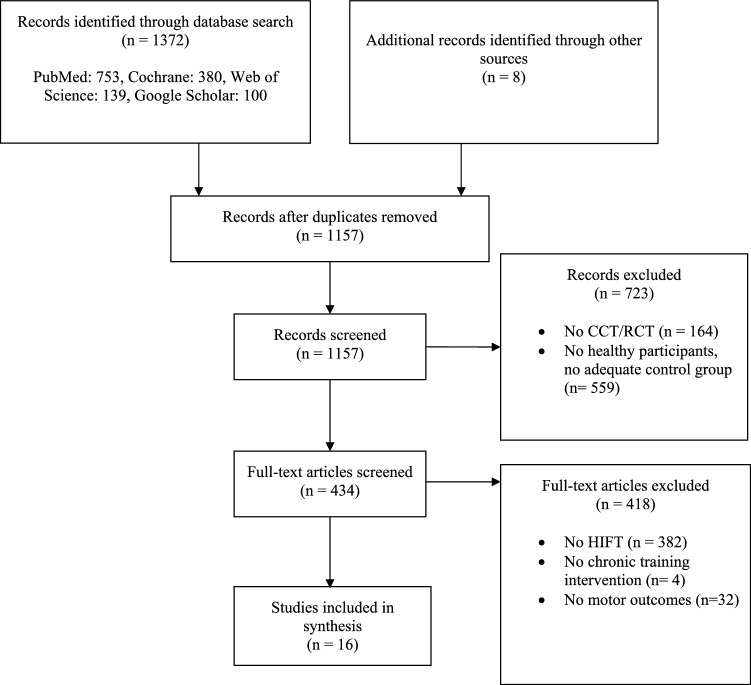
PRISMA chart of the study flow. *CCT* controlled clinical trial, *RCT* randomized controlled trial, *HIFT* high-intensity functional training.

A flow diagram of the literature search is displayed in Fig. 1. The algorithms used returned a total of 1372 records. Sixteen studies^5,26–40^ (Table 1) met the eligibility criteria and were included in the review.

**Table 1:** Characteristics of the included studies.

Study	Design	Participants	HIFT protocol/control	Duration	Outcomes
Engel et al.^26^	Parallel-group 1: HIFT(n = 10) 2: NEX (n = 10)	n = 20 healthy, moderately trained adults (10 females and 10 males; age 36.2 ± 11.1 years; BMI: 23.9 ± 3.7 kg/m^2^)	1: 2x/week, 30 min, eight whole-body exercises with suspension trainer (e.g. squats, burpees, jumping jacks, chest press, mountain climbers), 20 s all-out exercise, 10 s rest 2: –	8 weeks	Strength: LP, CP, PD, BE Endurance: V_max_
Engel et al.^27^	Parallel-Group 1: HIFT(n = 17) 2: NEX (n = 18)	n = 35 secondary school children (24 males, 11 females, 11.7 ± 0.3 years)	1: 4x/week, 6 min, circuit-like all-out whole-body exercise (e.g. planks, burpees, skippings), varying durations and breaks 2: –	4 weeks	Strength and endurance: push-ups, sit-ups, standing LJ, Lateral jumps, 20-m sprint, Balance: steps backwards Endurance: 6-min run
Schmidt et al.^28^	Parallel-Group 1: HIFT-7 (n = 32) 2: HIFT-14 (n = 28) 3: NEX (n = 36)	n = 96, active collage students, (53 females, 43 males, age:18–24 years) 1: 17 females, 15 males 2: 15 females, 13males 3: 21 females, 15males	1: 3x/week, 7 min, whole-body exercises (e.g. jumping jacks, wall sit, push-ups, abdominal crunch, step-up chair, squat), 30 s exercise, 10 rest 2: Identical to group 1 but with 14 min duration in weeks 5–8 3: –	8 weeks	Strength and endurance: HST, push-ups Endurance: VO_2_max
Ballesta-Garcia et al.^29^	Parallel-Group 1: HIFT (n = 18) 2: NEX (n = 18)	n = 36 females (67.8 ± 6.2 years) 1: Age: 66.3 ± 5.4 years 2: Age: 67.4 ± 5.7 years	1: 2x/week, variable duration/intensity, whole-body exercises (e.g. jumping jacks, squats, walking) 2: –	18 weeks	Strength: arm curl test, STS-30, HST Balance: 1LST, TUG Endurance: HR, 6MWT
Sperlich et al.^30^	Crossover (wait-control intervention = 1: NEX /HIFT1 (n = 12) 2: NEX/HIFT2 (n = 12)	n = 24 untrained adults (14 females, 10 males; 25 ± 5 years) 1: 7 females, 5 males 2: 7 females, 5 males	1: 1x/day, 6 min, whole-body exercise (e.g. burpees, leg levers, push-ups) 2: Identical to group 1 but 2x/day	8 weeks (4 weeks NEX/4 weeks HIFT) 1	Strength: push-ups, leg levers, burpees, one-legged squat, skipping Endurance: Vo_2_max
McRae et al.^31^	Parallel-Group 1: MCT (n = 7) 2: HIFT (n = 7) 3: NEX (n = 8)	n = 22 recreationally active females 1: 21.1 ± 2.8 years 2: 20.7 ± 1 years 3: 19.2 ± 0.9 years	1: 4x/week, 30 min, treadmill running at 85% HR_max_ 2: 4x/week, 8 × 20 s single exercise (e.g. burpees, jumping jacks, mountain climbers) 3: −	4 weeks	Strength: LE, LC, lateral PD, CP, push-ups, sit-ups, BE Endurance: Vo_2_max, Bruce protocol
Schaun et al.^32^	Parallel-Group 1: HIIT (n = 15) 2: HIFT (n = 12) 3: MCT (n = 14)	n = 41 recreationally active males (23.7 ± 0.7 years)	1: 3x/week, 8 × 20 s treadmill running at 130% of the velocity associated to VO_2_max, 10 s rest 2: 3x/week, 8 × 20 s, 4 calisthenics exercises (burpees, mountain climbers, squat, thrusts), 10 s rest 3: 3x/week, 30 min, treadmill running at 90–95% of the HR associated to the ventilatory threshold	16 weeks	Strength: CMJ, SJ, EMG signals of RF & VL
Schaun et al.^33^	Parallel-Group 1: HIIT (n = 15) 2: HIFT (n = 12) 3: MCT (n = 14)	n = 41 recreationally active males (23.7 ± 0.7 years)	1: 3x/week, 8 × 20 s treadmill running at 130% of the velocity associated to VO_2_max, 10 s rest 2: 3x/week, 8 × 20 s, 4 calisthenics exercises (burpees, mountain climbers, squat, thrusts), 10 s rest 3: 3x/week, 30 min, treadmill running at 90–95% of the HR associated to the second ventilatory threshold	16 weeks	Endurance: Vo_2_max
Evangelista et al.^34^	Parallel-Group 1: HIFT (n = 14) 2: MCT (n = 11)	n = 25 physically active participants (unknown sex and age)	1: 3x/week, 20 sets of 30 s all-out exercise, 30 s rest (jumping jacks, mountain climbers, burpees, squat jumps) 2: 3x/week, 25 min running (80% HR_max_)	6 weeks	Strength: sit-ups, push-ups
Garcia-Pinillos et al.^35^	Parallel-Group 1: HIFT (n = 47) 2: NEX (n = 43)	n = 90 active adults (64 females, 26 males; 72.8 ± 5.7 years)	1: 3x/week, 35–40 min, high-intensity circuit strength training combined with high-intensity interval endurance training as active recovery (e.g. medicine ball throws, farmer’s walk, sit to stand) 2: –	12 weeks	Strength: HST, STS-30 Balance: CoP
Menz et al.^36^	Parallel-Group 1: HIIT (n = 8) 2: HIFT (n = 7)	n = 15 moderately trained adults (25.6 ± 2.6 years) 1: 6 females, 2 males 2: 5 females, 2 males	3–4 sets; 8 × 20 s all-out exercise, 10 s rest 1: running HIIT 2: functional HIIT with body weight	4 weeks	Strength: push-ups, toes to bar, BJ, burpees Endurance: Vo_2_max, HR_max_, BLA_max_
Buckley et al.^37^	Parallel-Group 1: HIIT (n = 14) 2: HIFT (n = 14)	n = 28 recreationally active females (24.7 ± 5.4 years) 1: 24.3 ± 5.2 years 2: 25.1 ± 5.6 years	1: 3x/week, 6 sets of 60 s all out intensity rowing, 3 min rest 2: 3x/week, 6 sets of 60 s all out intensity workout (strength exercise (4–6 repetitions), accessory movement (8–10 repetitions), metabolic component), 3 min rest	6 weeks	Strength: squat, CP, DL, BJ Endurance: Vo_2_max, anaerobic power, anaerobic capacity, squat endurance
Greenlee et al.^38^	Parallel-Group 1: HIFT (n = 129) 2: NEX (n = 129)	n = 258 adults 1: 61 females, 66 males; 24.7 ± 5.6 years 2: 63 females, 66 males; 24.3 ± 5.7 years	1: varying frequency and volume, e.g. resistance band exercixes, rope skipping, high-intensity cardiorespiratory exercises, 2: –	16 weeks	Strength: Push-ups, towers, LJ Endurance: Vo_2_max, 1.5-mile run
Jimenez-Garcia et al.^39^	Parallel-Group 1: HIFT (n = 26) 2: MCT (n = 24) 3: NEX (n = 23)	n = 73 recreationally active adults (unknown sex) 1: 68.2 ± 3.0 years 2: 68.8 ± 6.0 years 3: 68.5 ± 6.3 years	1:2x/week, 4 intervalls of 4 min suspension training exercises at 90–95% HR_max_, 3 min active rest 2: same as in group one but with 50–70% HR_max_ 3: guidelines to encourage phyical activity	12 weeks	Strength: HST, TUG
Wilke et al.^5^	Parallel-Group 1: HIFT (n = 20) 2: MCT (n = 13)	n = 33 untrained adults 1: 11 females, 9 males; 24.5 ± 6.3 years 2: 10 females, 3 males; 23.7 ± 3.2 years	1: 3x/week, 15 min, whole-body exercises (e.g. squats, burpees, push-up), 20 s all-out exercise, 10 s rest 2: 3x/week, 50 min, walking at 50–60% HRR	6 weeks	Strength: SLHD, CMJ, RSI, LP, CP Balance: CoP Endurance: Vo_2_max
Islam et al.^40^	Parallel-Group 1: HIFT (n = 26) 2: MCT (n = 27) 3: NEX (n = 15)	n = 68 inactive adults, 51 females, 17 males, 21 ± 3 years	1: 4x/week, 4 × 20 s whole-body exercises (burpees, mountain climber, jumping jacks, sqaut) performed at 20 s intervals with 10 s rest 2: 4x/week, 30 min, running on treadmill at 85% HR_max_ 3: –	4 weeks	Endurance: VO_2_max, 5 km TT, TTF

Age data are means ± standard deviations.

*HIFT* high-intensity functional training, *MCT* moderate continuous training, *NEX* no exercise, *min* minutes, *s* seconds, *LP* leg press, *CP* chest press, *PD* pull downs, *BE* back extensions, *V*_*max*_ maximal running speed, *HRR* heart rate reserve, *HR* heart rate, *LJ* long jump, *HST* hand strength, *VO*_*2*_*max* maximal rate of oxygen consumption, *STS-30* 30 s. sit-to-stand, *TUG* timed up and go, *1LST* one leg stance, *6MWT* 6 min walking test, *LE* leg extensions, *LC* leg curls, *CMJ* counter movement jump, *SJ* squat jump, *EMG* electromyography, *RF* rectus femoris, *VL* vastus lateralis, *CoP* center of pressure, *BJ* broad jump, *BLA*_*max*_ maximal blood lactate concentration, *DL* dead lift, *SLHD* single leg hop distance, *RSI* reactive strength index, *TT* time trial, *TTF* time to fatique.

### Characteristics of the studies

The 16 papers collectively evaluated 864 participants (302 men and 458 women, distribution unknown for two studies) with mean ages ranging from 11.7 to 72.8 years (Table 1). Most trials (n = 15) used a parallel-group design while only one employed a crossover design.

Ten papers^26–31,35,38–40^ compared HIFT vs. NEX, four papers^32,33,36,37^ examined HIFT vs. HIIT, seven papers investigated HIFT vs. MCT^5,31–34,39,40^. No studies comparing HIFT vs. RES or BAL were found.

Complete data were available, obtained or extracted from figures for nine studies^5,31–33,36–40^, whilst imputation of standard deviations was needed for 7 trials^26–30,34,35^.

### Methodological quality

The two reviewers agreed in 155 (96.9%) of the 160 criteria scored by means of the PEDro scale. All disagreements were resolved by discussion. The methodological quality of the 16 included papers ranged from 4 to 7 out of 10 and their mean scores, in sum (5.9 ± 0.9), were classified as moderate. Most studies reported randomization, between-group comparisons and point estimates plus variability indicators, had comparable baseline values and used intention-to-treat. In contrast, only a few studies used concealed allocation as well as therapist/instructor, participant or assessor blinding (Table 2).

**Table 2:** Methodological quality of the studies included (ratings on the PEDro scale).

Study	Inclusion criteria^a^	Random allocation	Concealed allocation	Similarity at baseline	Subject blinding	Therapist blinding	Assessor blinding	> 85% follow-up	Intention to treat analysis	Between-group comparisons	Point estimates and variability	Total (points)
Engel et al.^26^	+	+	−	+	−	−	−	+	+	+	+	6
Engel et al.^27^	+	+	−	+	−	−	−	+	+	+	+	6
Schmidt et al.^28^	+	+	−	+	−	−	−	+	+	+	+	6
Ballesta-Garcia et al.^29^	+	+	−	+	−	−	+	−	+	+	+	6
Sperlich et al.^30^	+	+	−	+	−	−	−	+	+	+	+	6
McRae et al.^31^	−	−	−	+	−	−	−	+	−	+	+	4
Schaun et al.^32^	+	+	−	+	−	−	+	+	+	+	+	7
Schaun et al.^33^	+	+	−	+	−	−	+	−	+	+	+	6
Evangelista et al.^34^	+	+	−	+	−	−	−	+	+	+	+	6
Garcia-Pinillos et al.^35^	+	+	−	+	−	−	−	+	+	+	+	6
Menz et al.^36^	+	+	−	+	−	−	−	−	+	+	+	5
Buckley et al.^37^	−	+	−	+	−	+	−	+	+	+	+	7
Greenlee et al.^38^	+	+	−	+	−	−	−	−	−	+	+	4
Jimenez-Garcia et al.^39^	+	+	+	+	−	−	−	+	+	+	+	7
Wilke et al.^5^	+	+	−	+	−	−	−	+	+	+	+	6
Islam et al.^40^	+	+	−	+	−	−	−	+	+	+	+	6

^a^External validity, not counted for score, +  = point awarded,− = no point awarded.

### HIFT vs. no exercise

**Figure 2:**
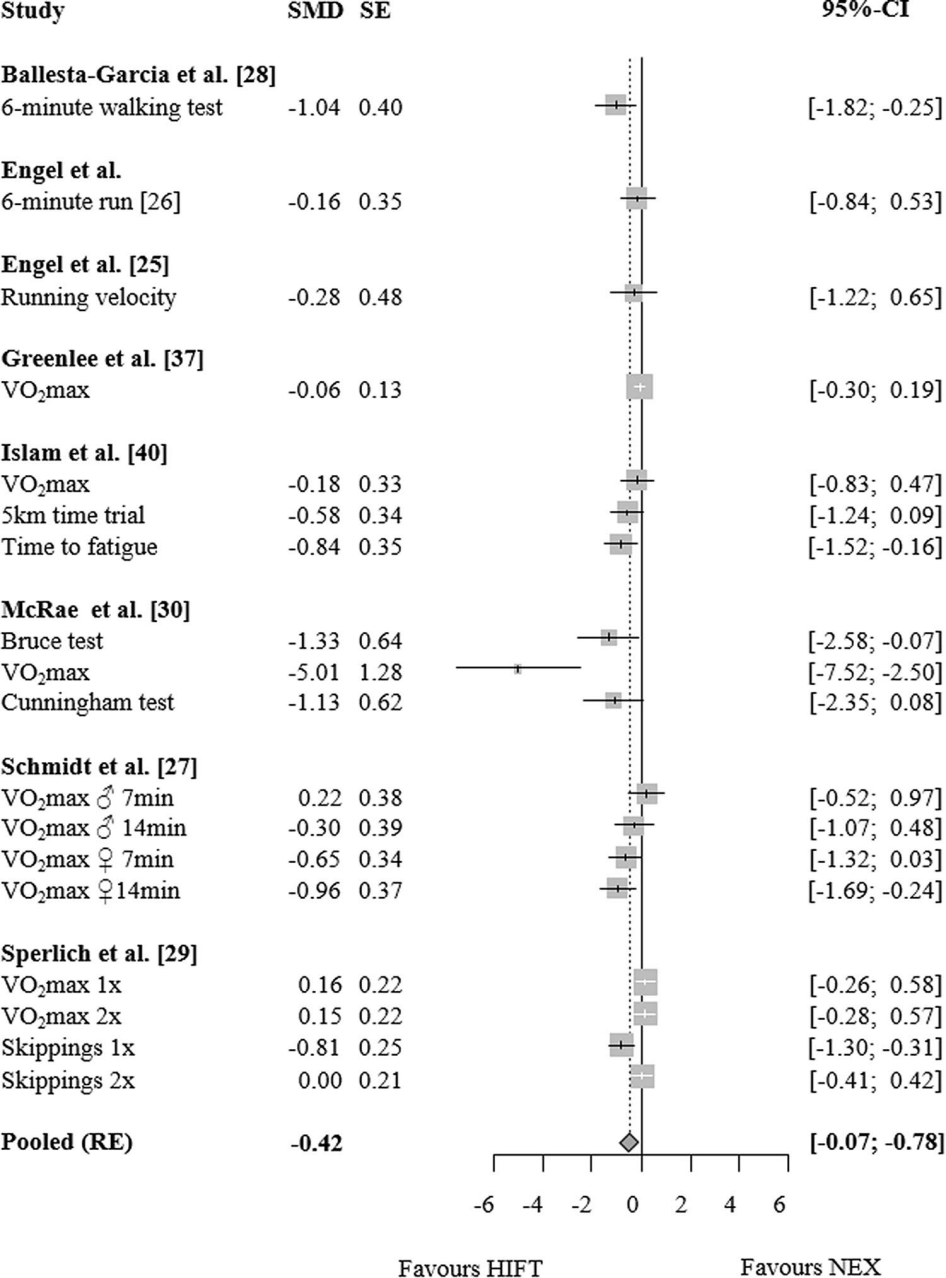
Effects of high-intensity-functional training (HIFT) vs. no exercise (NEX) on markers of endurance performance. Forest plots with pooled standardized mean differences (SMD), standard errors (SE) and 95% confidence intervals (CI) are displayed. *RE* random effects.

**Figure 3:**
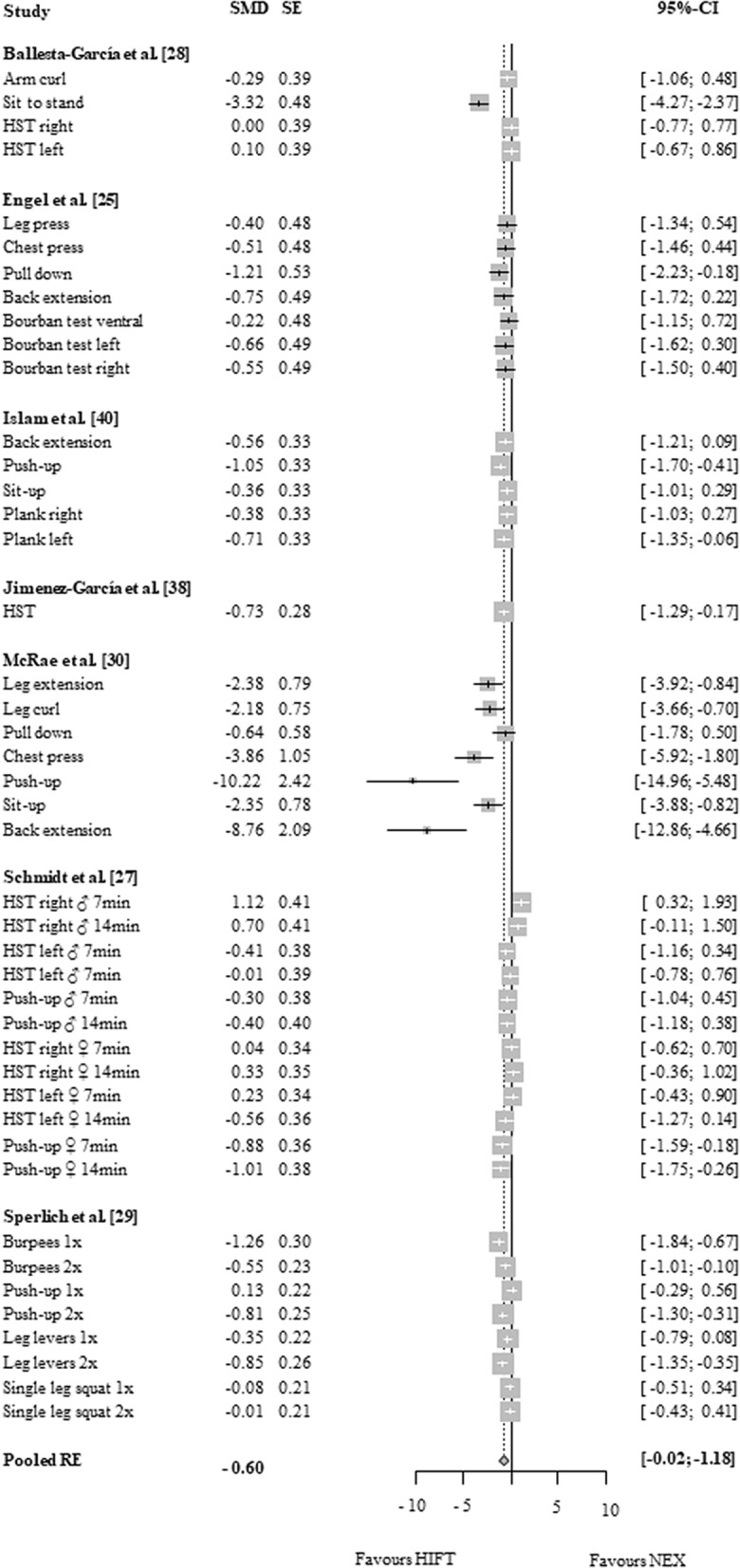
Effects of high-intensity-functional training (HIFT) vs. no exercise (NEX) on markers of muscle strength. Forest plots with pooled standardized mean differences (SMD), standard errors (SE) and 95% confidence intervals (CI) are displayed. *HST* hand strength, *min* minutes, *RE* random effects.

**Figure 4:**
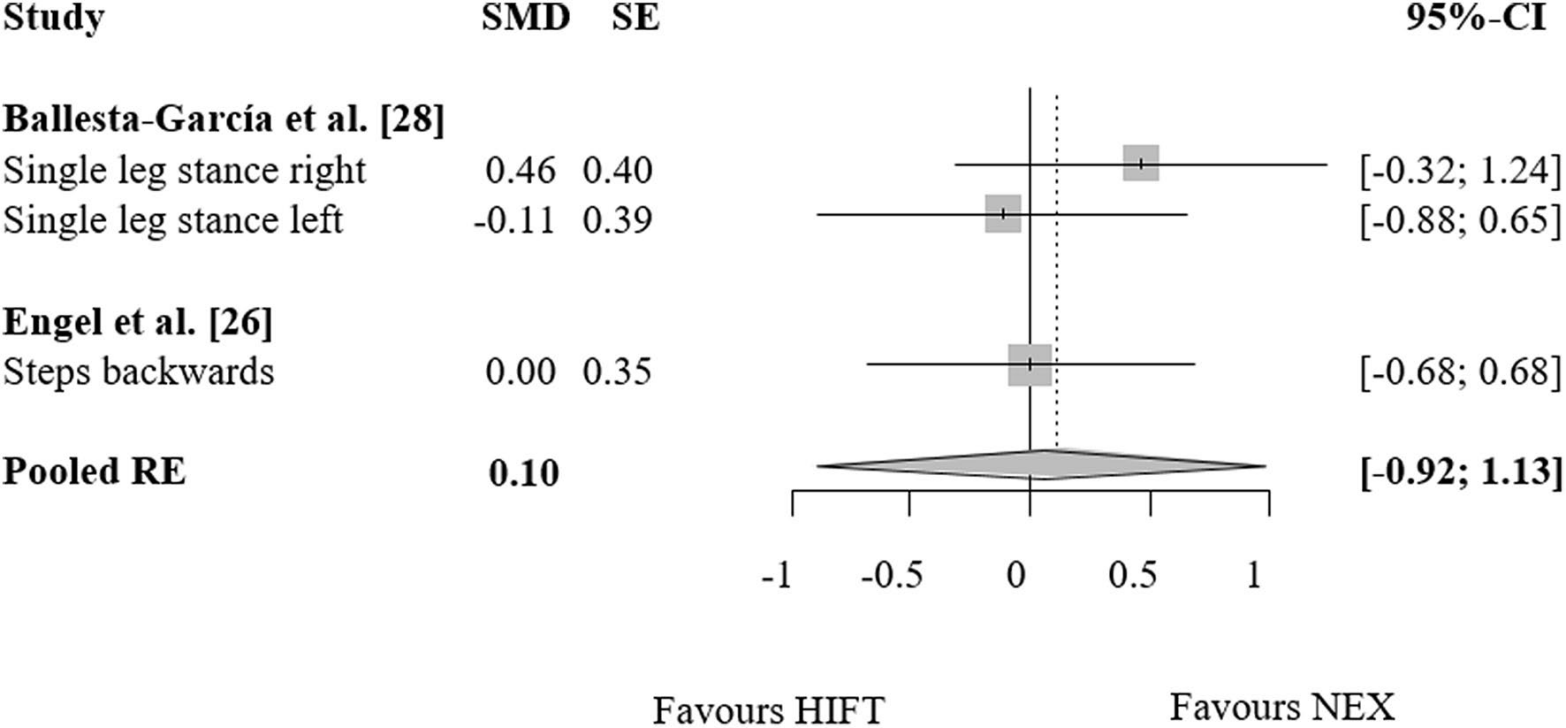
Effects of high-intensity-functional training (HIFT) vs. no exercise (NEX) on markers of balance. Forest plots with pooled standardized mean differences (SMD), standard errors (SE) and 95% confidence intervals (CI) are displayed. *RE* random effects.

For endurance capacity, a small ES favouring HIFT over NEX was found (SMD: 0.42, 95% CI: 0.07 to 0.78, p = 0.03, Tau^2^: 0.19, ω^2^: 0, n = 8 studies, n = 18 ES, Fig. 2). Similarly, a moderate-magnitude ES suggested superiority of HIFT regarding strength (SMD: 0.60, 95% CI 0.02–1.18, p = 0.04, Tau^2^: 0.50, ω^2^: 0, n = 7 studies, n = 44 ES, Fig. 3). In contrast to the above outcomes, only very few studies assessed balance. The pooled comparison did not yield a significant difference between HIFT and NEX (SMD: − 0.10, 95% CI − 1.13 to 0.92, p = 0.42, Tau^2^: 0, ω^2^: 0.01, n = 2 studies, n = 3 ES, Fig. 4).

In most cases (Table 3), no differences between the levels of the tested moderator variables were found (p > 0.05, Table 3). The only exeception was sex: Females appeared to respond more strongly to HIFT with regard to endurance capacity when compared to mixed samples (p = 0.02). A comparison against men only was impossible due to a lack of data. The same applied for the moderator analysis of HIFT vs. NEX regarding balance.

**Table 3:** Results of the moderator analysis.

Comparison	Moderator	No. of studies (ES)	Mean estimate (95% CI)	Tau^2^/omega^2^
**Endurance**				
HIFT vs. NEX	**Sex**			0.11/0
	Mixed	5 (10)		
	Female	3 (6)	− 0.88 (0.19 to 1.56)*	
	**Intervention duration (weeks)**			0.28/0
	Short (< 6)	4 (11)		
	Long (> 6)	4 (7)	− 0.05 (− 0.82 to 0.71)	
	**Session duration (min)**			0.28/0
	Short (< 7)	5 (15)		
	Long (> 7)	3 (3)	− 0.05 (− 0.82 to 0.71)	
	**Total program volume (min)**			0.17/0
	Low (< 168)	5 (11)		
	High (> 168)	4 (6)	− 0.33 (− 1.07 to 0.40)	
**Strength**				
HIFT vs. NEX	**Sex**			0.64/0
	Mixed	2 (17)		
	Female	4 (21)	0.50 (− 2.17 to 1.16)	
	**Age (years)**			0.55 /0
	Young (< 40)	5 (39)		
	Old (> 40)	2 (5)	0.21 (− 0.56 to 0.97)	
	**Intervention duration (weeks)**			0.55 /0
	Short (< 7)	5 (39)		
	Long (> 7)	2 (5)	0.21 (− 0.56 to 0.97)	
	**Session duration (min)**			0.60 /0
	Short (< 15)	4 (26)		
	Long (> 15)	4 (18)	− 0.19 (− 0.74 to 1.13)	
	**Total program volume (min)**			0.43 /0
	Low (< 168)	4 (22)		
	High (> 168)	4 (18)	− 0.23 (− 1.38 to 0.92)	
	**Interval duration (s)**			0.88/0
	Short (< 20 s)	4 (31)		
	Long (> 20 s)	2 (5)	0.12 (− 1.21 to 1.45)	
	**Break duration (s)**			0.88/0
	Short (< 10 s)	4 (31)		
	Long (> 20 s)	2 (5)	0.12 (− 1.21 to 1.45)	

*HIFT* high-intensity functional training, *NEX* no exercise, *HIIT* high-intensity interval training, *MCT* moderate continuous training, *no* number, *ES* effect size, *CI* confidence interval.

Asterisks mark statistical significance of a moderator level (p < 0.05).

### HIFT vs. endurance training

**Figure 5:**
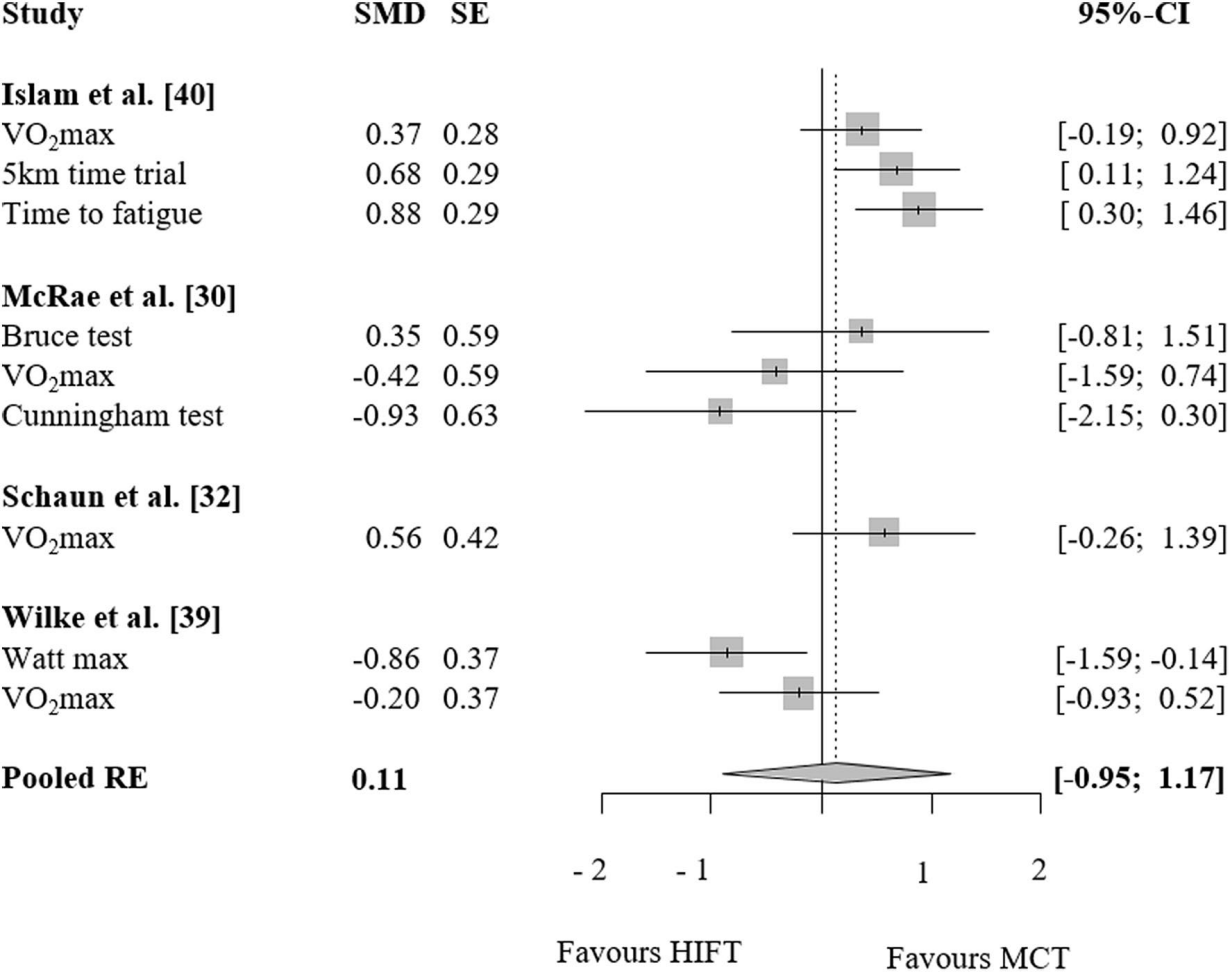
Effects of high-intensity-functional training (HIFT) vs. moderate continuous aerobic training (MCT) on markers of endurance. Forest plots with pooled standardized mean differences (SMD), standard errors (SE) and 95% confidence intervals (CI) are displayed. *RE* random effects.

**Figure 6:**
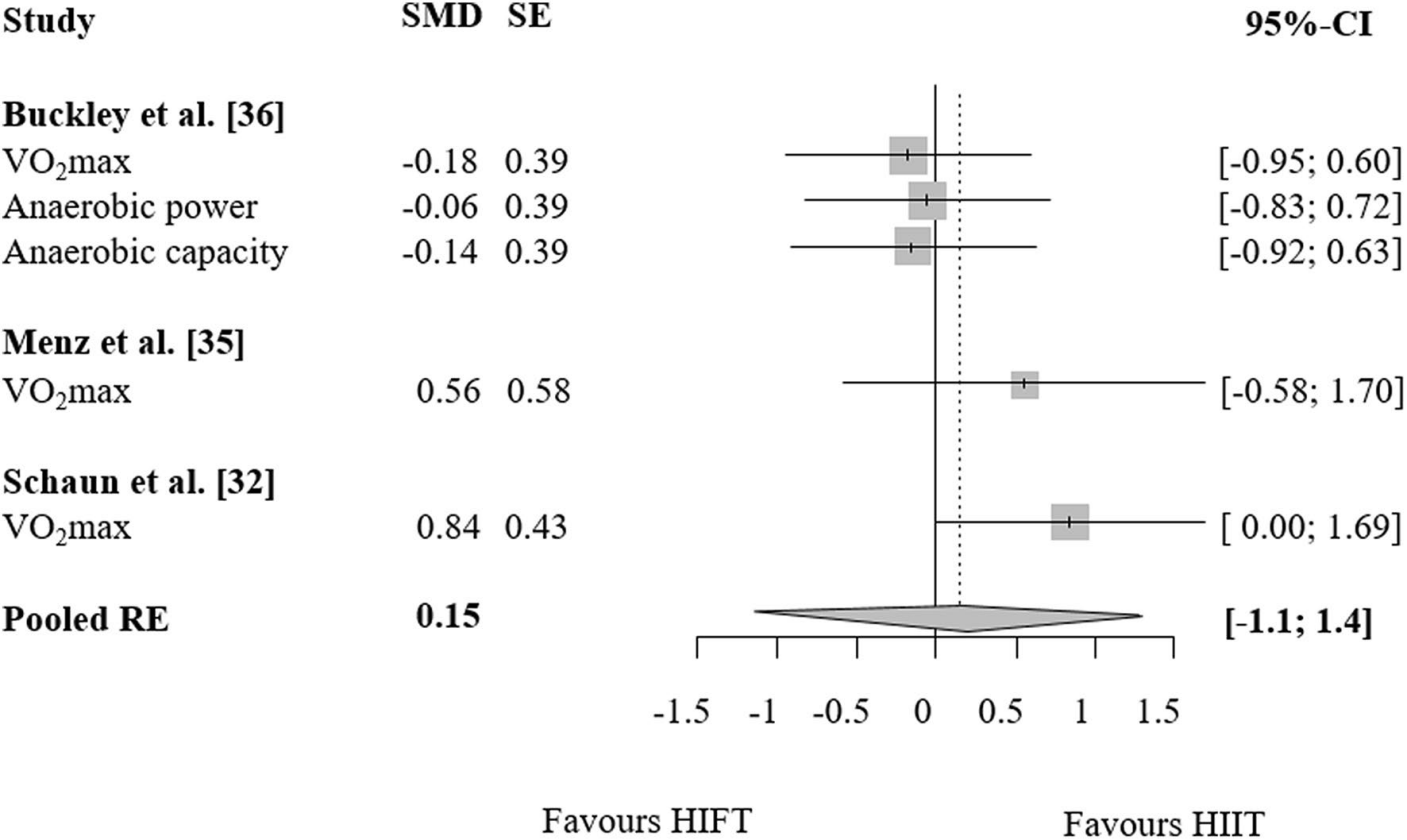
Effects of high-intensity-functional training (HIFT) vs. high-intensity interval training (HIIT) on markers of endurance. Forest plots with pooled standardized mean differences (SMD), standard errors (SE) and 95% confidence intervals (CI) are displayed. *RE* random effects.

Meta-analytic pooling did not reveal any differences between HIFT and MCT with regard to endurance capacity (SMD: − 0.11, 95% CI − 1.17 to 0.95, p = 0.75, Tau^2^: 0.29, ω^2^: 0.06, n = 4 studies, n = 9 ES, Fig. 5). Similarly, no differences were found for HIFT vs. HIIT (SMD: − 0.15, 95% CI − 1.4 to 1.1, p = 0.66, Tau^2^: 0.048, ω^2^: 0, n = 3 studies, n = 5 ES, Fig. 6). Due to the small number of studies, data were insufficient for moderator analyses.

### Risk of bias

**Figure 7:**
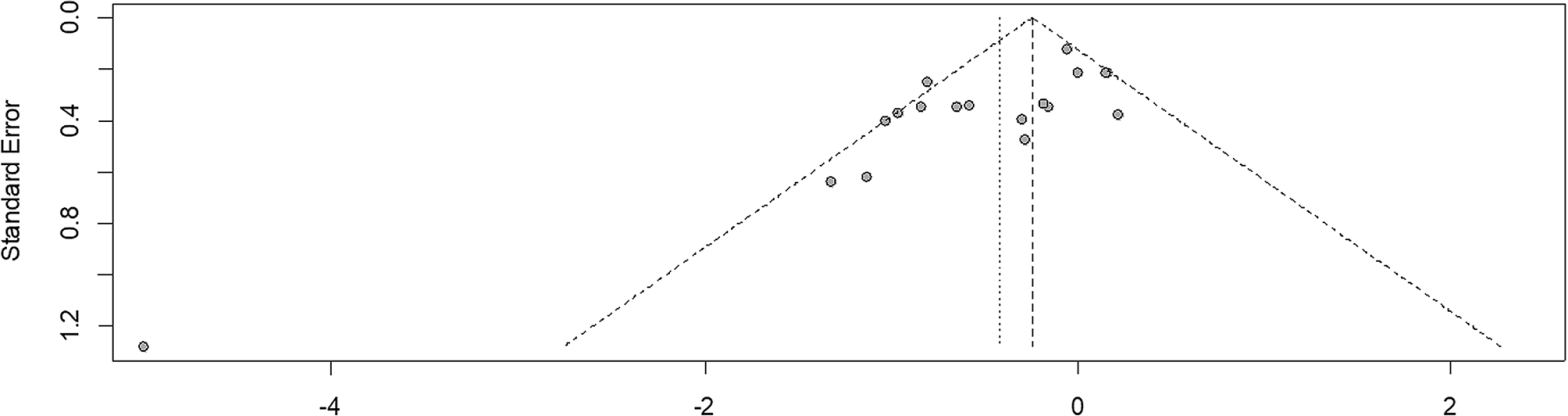
Funnel plot of the effect of high-intensity functional training vs. no-exercise (effect size against standard error). Note the the outlier on the lower left.

Visual inspection of the funnel plots (Fig. 7 for an example) suggested a potential reporting bias for HIFT vs. NEX regarding both, endurance and muscle strength markers due to a large standard error and small sample size in few studies. Sensitivity analyses without these trials, however, did not modify the general conclusions. Due to small numbers of ES (< 10), reporting bias could not be assessed for HIFT vs. MCT and HIFT vs. HIIT.

## Discussion

This systematic review with meta-analysis presents the first differentiated summary of the long-term effects of HIFT on markers of motor performance. Our results demonstrate that related interventions, performed over multiple weeks, induce small to moderate improvements in endurance and strength capacities. Regarding the former, HIFT is neither superior nor inferior to traditional exercise regimes such as aerobic training or HIIT.

The mechanisms by which HIFT acts on the body are a matter of debate. The performance of repeated whole-body exercises provokes a cardioplumonary output which is comparable to traditional endurance training: Tibana et al.^41,42^ found maximum heart rates of more than 180 beats per minute and blood lactate concentrations of 11–18 mmol/l following two HIFT workouts. It is therefore plausible that repeated engagement in HIFT improves endurance capacity. In resistance training, time under tension represents an essential parameter steering protein synthesis^43^. One hallmark of HIFT is the execution of high repetition numbers with short breaks^2^. As time under tension is rather long, this may create a potent stimulus. High metabolic stress represents another important trigger for muscle hypertrophy. After acute bouts of HIFT, elevated pro-inflammatory cytokines (IL-6) were observed^41^. This, in concert with the high blood lactate levels, may lead to metabolic conditions which are favourable for muscle adaptations.

The findings of our meta-analysis have significant implications for health professionals and fitness coaches. Firstly, based on the available evidence, HIFT appears to represent a viable alternative to conventional training methods if clients and patients do not like traditional resistance or endurance training. Secondly, besides general aversion towards sport or specific exercise regimes, many inactive individuals state lack of time as a main brarrier to engage in physical activity^3,4^. As HIFT can be performed at short overall durations and, contrary to HIIT, seems to address multiple motor abilities, it represents an intriguing option when aiming to increase participation in and adherence to physical acivity programs. This particularly applies because formerly inactive persons reported higher levels of exercise enjoyment following HIFT when compared to the often prescribed conventional endurance training^5^.

Despite the promising fields of application, several aspects call for further research. In our review, we focused on markers of strength, endurance and balance, which are of interest for most members of the normal population. However, athletes, in addition, frequently require capacities such as speed or power and hence, it would be intriguing to examine the potential effect of HIFT in this context too. Also, while various studies compared HIFT to NEX as well as HIFT to different types of endurance exercise, there is a lack of trials regarding the comparison against resistance training and balance training. Upcoming studies should hence aim to incorporate these two motor capacitities. Another issue relates to methodological considerations. Inspection of the funnel plots suggested the possibility of a publication bias and hence the true effect sizes may be slightly different to the ones reported here. Although this should be underlined when interpreting the results, for several reasons, funnel plot asymmetry must not be overestimated. In general, it has been shown that a non-normal shape of the graph represents a hint but not necessarily a proof of presence of bias as it can also be the result of between-study heterogeneity, small-sample study quality, chance and the use of standardized mean differences^44,45^. As our sub-analyses excluding outliers did not modify the main result of the analysis, we conclude that no substantial reporting bias is present. Regarding effect modifiers, with our moderator analysis, we made a strong effort to test the relevance of a plethora of variables. Yet, some others remain to be examined. Only four of the identified studies included older participants and only one was performed in children. It would be reasonable to assume that the treatment response substantially differs as a function of age and hence there is a need to design future trials for children and elderly persons. In a similar way, sex is another interesting but understudied parameter. While many samples were mixed or consisted of women, only two studies exclusively recruited males. In view of the hormonal differences (i.e. the higher testosterone levels in men), it can be assumed that particularly strength gains will be different depending on the sex of the participants^46^.

## Conclusions

HIFT represents an effective method to increase muscle strength and endurance capacity. In view of the multidimensional adaptations and the relatively small time effort, it may, therefore, be of value for both active and inactive individuals. Despite these promising findings, the factors moderating the treatment response remain largely obscure. In this context, future studies may specifically focus on the potential roles of age and sex.

## Data Availability

The datasets generated during and/or analysed during the current study are available from the corresponding author on reasonable request.
